# Investigation on the Vibration Induced by the Rotary-Shaft-Seal Condition in a Centrifugal Pump

**DOI:** 10.3390/s25175399

**Published:** 2025-09-01

**Authors:** Jiamin Zou, Yin Luo, Yuejiang Han, Yakun Fan, Chao Wang

**Affiliations:** 1 Research Center of Fluid Machinery Engineering and Technology, Jiangsu University, Zhenjiang 212013, China; 2 Wenling Research Institute of Fluid Machinery, Jiangsu University, Taizhou 318000, China; 3 Tianjin Fire Research Institute, Tianjin 300381, China; 4 Department of Mechanical and Mold Engineering, Taizhou Vocational College of Science and Technology, Taizhou 318020, China

**Keywords:** centrifugal pump, rotary shaft-seal, vibration, EMD-AR spectrum, failure analysis

## Abstract

During operation, failures in a centrifugal pump’s rotary shaft seal—such as wear, deformation, or thermal cracking—can adversely affect system performance. This study utilizes both theoretical and experimental methods to investigate the vibration characteristics of centrifugal pumps under different rotary-shaft-seal conditions. Vibration signals are collected and processed using empirical mode decomposition (EMD) and autoregressive (AR) modeling to generate an EMD-AR spectrum. The results show that rotary-shaft-seal failure leads to decreases in both the head and efficiency of the centrifugal pump. For improved operation stability, centrifugal pumps should operate at or slightly above their design flow rates (*Q_d_*), while avoiding low-flow conditions. Furthermore, the amplitude of the EMD-AR spectrum increases progressively as rotary-shaft-seal degradation worsens. Therefore, the EMD-AR spectrum provides a reliable diagnostic indicator for detecting rotary-shaft-seal damage.

## 1. Introduction

Centrifugal pumps are one of the most commonly used mechanical devices in industry applications that convert mechanical energy into hydraulic energy for fluids [1]. A centrifugal pump achieves optimal performance at its design flowrate (*Q_d_*), where the head (*H*), flow rate (*Q*), and rotational speed (*n*) are balanced [2]. Due to their high efficiency and operational stability, centrifugal pumps are extensively applied in key industries such as chemical processing, aerospace, agricultural irrigation, wastewater treatment, thermal power generation, and wind energy production [3].

Rotary shaft seals are standard components in most centrifugal pumps [4]. Failures related to rotary shaft seals account for more than 40% of all mechanical failures [5]. Fluctuations in fluid-induced excitation forces may destabilize the operation of centrifugal pumps [6,7], leading to reduced efficiency. Proper functioning of the rotary shaft seal is critical for pump reliability. Therefore, it is essential to establish suitable monitoring indicators to detect rotary-shaft-seal faults and ensure safe system operation.

At present, extensive research on rotary-shaft-seal fault monitoring has been conducted. Rotary-shaft-seal conditions are commonly evaluated using indicators based on pressure [8], stator current [9,10,11], acoustics [12,13], and vibration signals. Zhang [8] adopted a fault identification method based on a support vector basis to realize the pattern recognition of hydraulic reciprocating seal status; Chen [9] collected signals by adding an eddy current sensor to determine the size of the mechanical seal end face gap; Chen [10] realized the fault detection of a mechanical seal by collecting the pump motor current signal and the changes of the sealing gap liquid; Xia [12] analyzed the operating status of the mechanical seal end face through acoustic emission signals to achieve fault diagnosis of gas seals; Anderson et al. [13] proposed a method to detect the contact state of the end faces of liquid-lubricated mechanical seals using ultrasonic sensors to detect mechanical seal failures.

Vibration-based monitoring methods are the most widely applied [14]. Mechanical failures are often detected by increases in the amplitude of characteristic frequency components [15]. It is reported that approximately 80% of mechanical faults manifest in the vibration signals. Therefore, early analyses of these signals are of significant practical importance [16,17,18,19,20]. Hodkiewicz et al. [21,22,23] applied time–frequency and wavelet analysis methods to analyze the effect of different operating conditions on the vibration behavior of bearing seats in industrial double-suction pumps. Lu et al. [24] reported that the inlet attack angle of impeller blades significantly influences flow instability in centrifugal pumps. To address this, they proposed the Flow State Identification with Vibration (FSIV) method for detecting flow instabilities. Experimental results demonstrated that their method can effectively detect rotating stall, hump, backflow, unstable flow, and cavitation in centrifugal pumps, thereby contributing to safer and more stable system operation. Chen et al. [25] employed the LMS Test Lab system to measure shell vibration, shaft vibration, and the shaft center trajectory of pump assemblies under varying flow conditions and rotational speeds. Lin et al. [26] analyzed the pressure pulsation and energy characteristics of the guide vanes of an axial flow pump based on the Hilbert–Huang transform method and considered the impeller–guide vane interaction. Krzysztof et al. [27] performed transient diagnosis of hydraulic systems based on short-time Fourier transform. Yang et al. [28] analyzed the time-frequency signal of flow pulsation in a siphon outlet pipe based on the HHT method considering the pump–pipe interaction.

Currently, extensive research focuses on operating conditions monitoring and internal impeller damage detection for centrifugal pumps, while the more frequent failures of rotary shaft seals are often overlooked. Few studies have investigated the operational behavior of rotary shaft seals or extracted their fault-related features. Existing methods for diagnosing rotary-shaft-seal faults primarily rely on pressure, eddy current, and acoustic methods for detection and estimation. However, acquiring these signals generally requires high-precision sensors, which are relatively expensive and require invasive installation on the pump body, disrupting the centrifugal pump structure and making the sensors susceptible to damage. This study utilizes an accelerometer to collect vibration signals. This sensor only needs to be attached to the pump body, which does not disrupt the system structure, making it convenient and fast. Vibration signals offer advantages in early detection, real-time response, and parallel diagnosis of multiple faults, with high reliability. This study introduces a novel approach to analyze the condition of rotary shaft seals using vibration signals. Indicators are developed based on the characteristic frequencies of vibration signals to enable fault diagnosis of rotary shaft seals in centrifugal pumps.

## 2. Theory

### 2.1. Faults in Rotary Shaft Seals

The operation of a rotary shaft seal depends on the close contact between two highly flat surfaces, forming a sealing interface that effectively minimizes the risk of leakage. One surface remains stationary, while the other rotates with the shaft, enabling dynamic sealing during operation. The contact area where the two sealing rings meet is referred to as the sealing end face. Based on the type of host equipment, rotary shaft seals can be categorized for use in pumps, reactors, piston compressors, fans, refrigerators, and other machinery. Functionally and structurally, rotary shaft seals are generally divided into two categories: sliding types (push-ring type) and non-sliding types (bellows or corrugated pipe type). The primary distinction between these two types lies in whether the auxiliary sealing ring of the compensating ring allows axial relative movement.

In general, the rotary shaft seal is considered a weak point in fluid machinery, with its failure being one of the primary causes of equipment maintenance. Rotary-shaft-seal failures can generally be attributed to the following causes: dry running, chemical attack, cavitation, excessive heat, improper installation, shaft movement due to bearing wear, worn shafts or components, and the ingress of solid particles. Chemical and thermal damage are the most prevalent failure modes: (1) Chemical damage: Every material has specific vulnerabilities. Incompatibility between the sealing materials and the working fluid can result in issues such as cracking, swelling, shrinkage, or degradation of internal components, including rubber seals, gaskets, impellers, pump casings, and diffusers. (2) Thermal damage: Elevated fluid temperatures can cause elastomers to swell or melt due to excessive heat, reducing their sealing effectiveness and increasing the risk of leakage. This study focuses on the wear of rotary-shaft-seal end faces, which results from variations in the thickness of the liquid film between the sealing surfaces.

**Figure 1:**
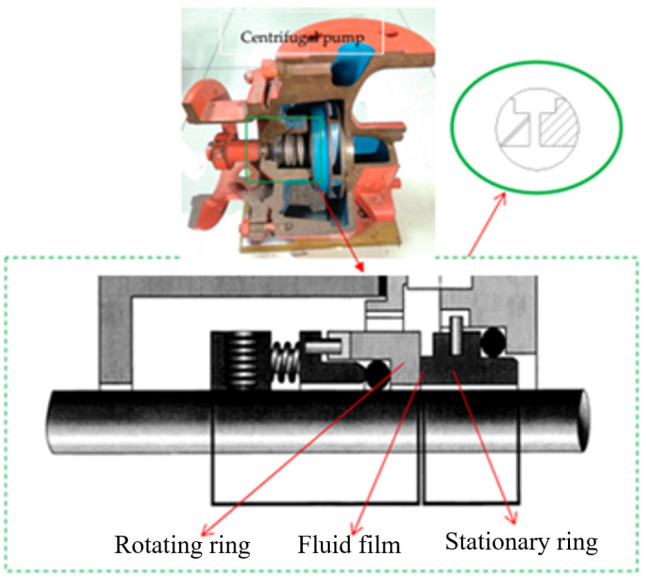
Structure of a rotary shaft seal.

Figure 1 illustrates the structural configuration of a rotary shaft seal. It consists of two circular components, the rotating ring and the stationary ring, which interact to form the sealing interface. A stable fluid film is maintained between the two rings. This fluid film ensures a minimal and controlled separation between the rings, effectively preventing dry friction and reducing wear.

The optimal operating condition of a rotary shaft seal is achieved when its performance meets the design and operational requirements of the system. It is important to note that rotary shaft seals do not necessarily ensure zero leakage between the sealing surfaces [10]. System pressure generates resistance that suppresses liquid leakage. This resistance also maintains a lubricating film on the sealing faces, thereby improving sealing effectiveness and reducing wear.

**Figure 2:**
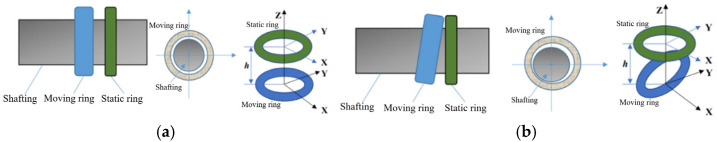
The state of a rotary shaft seal: (**a**) standard and (**b**) faulty condition.

As shown in Figure 2a, the model depicts the rotary shaft seal operating under normal conditions, where a consistent gap between the sealing surfaces ensures a uniform and stable liquid film in terms of both thickness and geometry. During operation, the rotor system maintains balanced axial and radial forces. Under these conditions, no contact or friction occurs between the rotating and stationary rings of the shaft seal. The flow within the system remains relatively stable, preventing abrupt fluctuations in hydraulic load torque.

Figure 2b presents a simplified model illustrating the failure state of a rotary shaft seal. Such failure has significant consequences for pump operation, resulting in unbalanced forces within the shafting system. A sudden increase in radial force causes elevated friction between the rotating ring and the shaft, while changes in axial force alter the liquid film thickness between the sealing rings, thereby increasing friction at the sealing interfaces. Overall, the failure of the rotary shaft seal induces rotor imbalance and fluid force asymmetry at the sealing end faces. These effects lead to torque fluctuations, increased vibration, and further deterioration of system stability.

**Figure 3:**
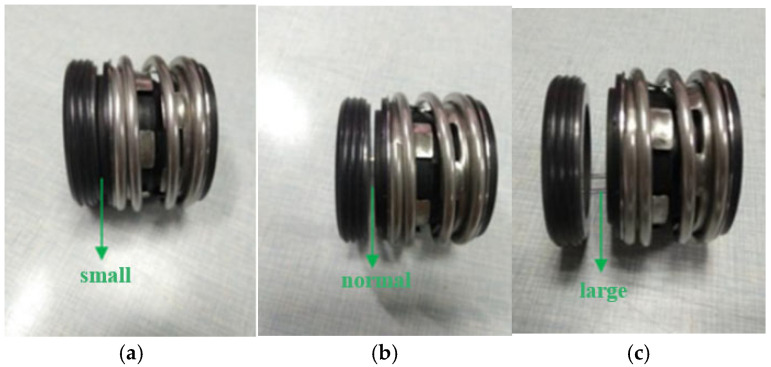
Different degrees of extrusion failure and schematic diagram: (**a**) small end-face clearance; (**b**) normal state; and (**c**) large end-face clearance.

During operation, the end face of the rotary shaft seal is subject to compression and wear, which is one of the most common causes of seal failure [29]. Figure 3 illustrates failure modes of the rotary shaft seal under varying extrusion conditions. Based on the basic theory of mechanical seals, industry standards, and engineering practice, the normal clearance is generally 1–3 μm, with a small clearance defined as less than 1 μm and a large clearance defined as greater than 5 μm. Rotational deviation and misalignment of the seal ring end face destabilizes the liquid film, leading to uneven force distribution across the sealing surfaces and significant fluctuations in axial and radial forces.

### 2.2. Signal Preprocessing

**Figure 4:**
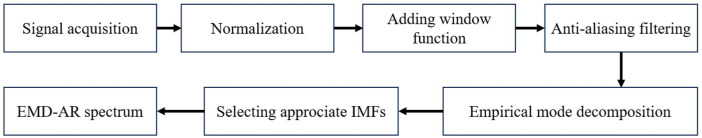
Signal preprocessing flowchart.

Common mechanical faults—such as rotational imbalance, eccentricity, shaft bending, and looseness of components—can often be directly identified from the vibration signal, making the diagnostic process relatively straightforward. However, more complex faults—including shaft damage, gear failure, bearing defects, and electrical faults—require further vibration signal analysis to achieve accurate diagnosis. Based on previous research, this study analyzes the vibration signal characteristics of centrifugal pumps under various operating conditions and fault scenarios [20]. Figure 4 presents the processing flow of vibration signals used for fault analysis.

Faults in rotary shaft seals lead to significant fluctuations in the forces acting on the rotating and stationary rings, which can disrupt flow conditions within the centrifugal pump and ultimately result in torque fluctuations in the shaft system [30]. The changes in Torque variation intensifies system vibration, making vibration signal analysis a valuable tool for fault examination. The vibration signal preprocessing steps in this study primarily include normalization, application of a window function, and anti-aliasing filtering, as detailed below:

(1) Normalization:

Normalization is used to scale the data to a defined range, typically between −1 and +1. This step reduces the influence of differing magnitudes across features, thereby enhancing model convergence speed and accuracy. Normalized data within the range of −1 to +1 also facilitates data visualization and analysis by allowing more intuitive observation of data distribution and characteristics.

(2) Windowing:

In Fast Fourier Transform (FFT) analysis, truncating an aperiodic signal introduces spectral leakage, resulting in trailing effects in the frequency spectrum. This occurs because truncation disrupts the signal’s periodicity, causing discontinuities at the boundaries. To minimize spectral leakage, a window function is applied to the signal prior to transformation, providing a weighted taper that smooths discontinuities [31]. The choice of window function influences the suppression of undesired frequency components and affects the trade-off between main-lobe width and sidelobe amplitude. Therefore, the window function must be selected based on the specific requirements of the signal processing task.

(3) Anti-Aliasing Filtering:

In practical applications, signals may contain frequency components higher than half the sampling frequency (the Nyquist frequency), which can lead to aliasing. While increasing the sampling rate can help reduce aliasing, it is not always feasible due to hardware limitations or data volume concerns. Therefore, an anti-aliasing filter—typically a low-pass filter—is applied before sampling to eliminate frequency components above the Nyquist limit, thereby preventing aliasing. This process prevents aliasing artifacts and minimizes the interference from unwanted high-frequency components [32]. The parameters of the anti-aliasing filter should be configured based on the specific characteristics of the signal and the sampling system.

### 2.3. Theories Related to Time–Frequency Joint Analysis

This paper proposes a feature extraction method that combines Empirical Mode Decomposition (EMD) with an Autoregressive (AR) model to analyze unsteady vibration signals under various rotary-shaft-seal conditions. Some basic concepts involved in time–frequency joint analysis are introduced in detail as follows.

(1) Instantaneous frequency:

A single-component signal contains only one frequency at any given moment, referred to as the instantaneous frequency of the signal. In contrast, a multicomponent signal comprises several instantaneous frequencies that vary over time. According to Ville’s definition, for a signal expressed as *x*(*t*) = *a*(*t*)cos∅(*t*), the instantaneous frequency is given by the following:

(1) $$f(t)=\frac{1}{2\pi }\frac{d\theta }{dt}$$

where *θ*(*t*) is the signal phase. Instantaneous frequency accurately characterizes only time-varying single-component signals and is generally unsuitable for most real-world signals [33]. The intrinsic mode function (IMF), as defined in the Hilbert–Huang Transform (HHT), satisfies the following two conditions: ① Over the entire data sequence, the number of extrema and zero crossings must either be equal or differ by at most one; ② At any point in time, the mean value of the upper envelope (formed by local maxima) and the lower envelope (formed by local minima) is zero—i.e., the envelopes are locally symmetric about the time axis [33].

**Figure 5:**
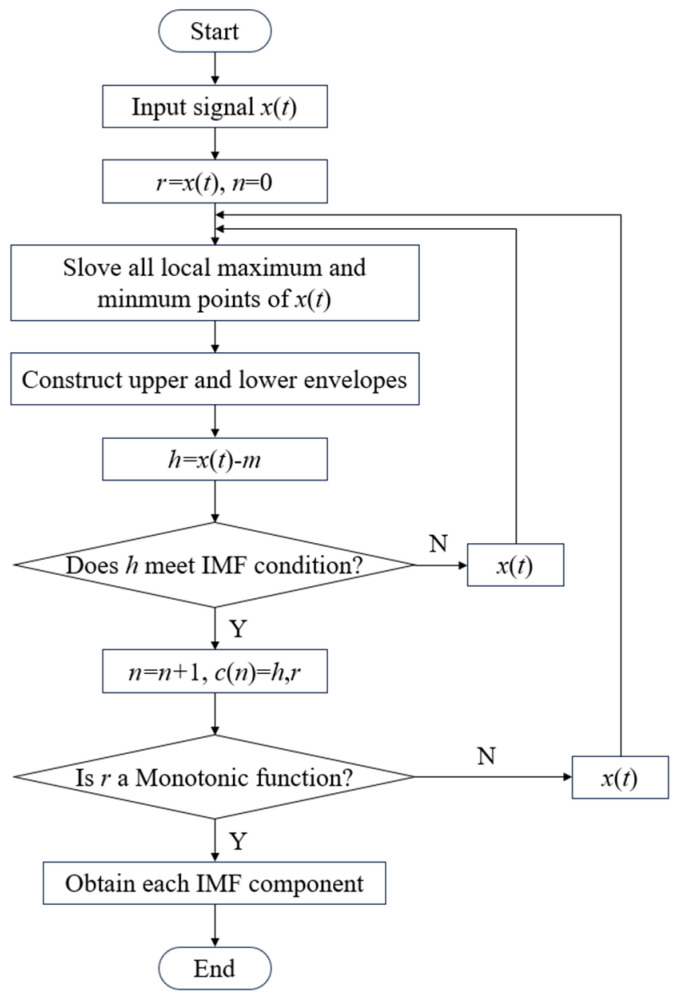
Algorithm flowchart to acquire IMFs.

(2) EMD decomposition principle:

The algorithmic procedure for decomposing complex signals into intrinsic mode functions (IMFs) is illustrated in Figure 5. For a given signal *x*(*t*), the empirical mode decomposition (EMD) process proceeds as follows [34]:

① Identify all local maxima and minima within the signal.
② Apply the cubic spline function for interpolating all maximum and minimum points, fitting both the upper and lower envelopes, and calculating the average value (*m*_1_) of these envelopes. Subsequently, perform the necessary calculations:

(2) $$h_{1}=x(t)-m_{1}$$

③ If *h*_1_ satisfies the conditions of an Intrinsic Mode Function (IMF), it is designated as the first IMF component of *x*(*t*). If *h*_1_ is not an IMF, repeat the sifting process *k* times using the current data as the new input, until an IMF is obtained. The final result is the following:

(3) $$h_{1k}=h_{1(k-1)}-m_{1k}$$

If *h*_1*k*_ satisfies the IMF criteria, it is identified as the first-order intrinsic mode function, denoted as *c*_1_.

④ Subtract *c*_1_ from *x*(*t*) to get the residual:

(4) $$r_{1}=x(t)-c_{1}$$

Repeat steps ①–③ using *c*_1_ as the input signal to extract the second component *c*_2_. This iterative process continues until the residual *r*_*n*_ becomes a monotonic function, at which point no further IMF components can be extracted, concluding the decomposition and yielding *n* intrinsic mode functions.

(5) $$x(t)=\sum_{i=1}^{n}c_{i}+r_{n}$$

EMD separates signal components based on characteristic time scales, from the shortest to the longest. The resulting IMFs satisfy the properties of completeness and near-orthogonality. Since EMD does not rely on a predetermined basis, the decomposition process is fully adaptive to the signal characteristics. The time-varying instantaneous amplitude and instantaneous frequency enhance decomposition efficiency and render EMD particularly effective for analyzing non-stationary and nonlinear signals [33].

(3) Guidelines for determining the IMF components

Due to inevitable human interference during cubic spline fitting, the mean value of the envelope in the actual IMF component may deviate from zero. Increasing the number of sifting iterations gradually reduces this mean, bringing it closer to zero; however, excessive sifting may distort the original signal’s intrinsic characteristics. Therefore, the standard deviation (SD) between two consecutive sifting results is used as a stopping criterion for the sifting process:

(6) $$SD=\sum_{t=0}^{T}\frac{{\left[h_{1(k-1)}-h_{1k}\right]}^{2}}{h_{1(k-1)}^{2}}$$

The number of iterations is controlled by the magnitude of the standard deviation (SD). Empirical evidence suggests that a SD threshold between 0.2 and 0.3 optimally balances the linearity and stability of the IMF while preserving its physical significance [33].

(4) EMD-AR spectrum

The AR model is a fundamental and widely used mathematical tool in time series analysis. In addition to capturing the characteristics and operational states of a system, it also offers extensibility for modeling observed data. This versatility makes the AR model valuable for both fault diagnosis and early fault prediction [35]. Moreover, AR model parameters can be used for power spectral estimation, significantly improving frequency resolution and enabling effective analysis of short-duration signals. Additionally, the resulting smoother spectrum facilitates distinction between different operating conditions, thereby enhancing fault feature extraction [33].

The AR model is primarily used for stationary processes. EMD separates non-stationary vibration signals into multiple IMFs, each with a zero mean and local symmetry about the time axis. This process effectively linearizes and stabilizes the original signal. Consequently, combining EMD with AR spectral analysis yields more effective analytical results. AR spectral estimation involves two primary steps. First, the AR model is constructed based on the time series signal. Second, the model coefficients are used to calculate the signal’s auto-power spectrum. The detailed steps are as follows:

① Establish the excitation–response characteristics of the system by generating a stationary signal sequence *x*(*n*) from a white noise sequence *u*(*n*).
② Estimate the parameters of the system *H*(*z*) using the known signal sequence *x*(*n*) or its autocorrelation function.
③ Estimate the power spectrum $P_{x}(k)$ of the sequence *x*(*n*) based on the parameters of *H*(*z*).

The general expression for the AR model is:

(7) $$x(n)=u(n)-\sum_{k=1}^{N}a_{k}x[n-k]$$

If Equation (7) is regarded as the input/output equation of the system, *u*(*n*) can be considered as the white noise input of the system and *x*(*n*) as the system’s output response to a finite-bandwidth white noise excitation.

According to the definition of the auto-spectrum, the one-sided power spectrum of the signal can be obtained using the system’s transfer function:

(8) $$G_{y}(f)=\frac{2T_{s}\sigma_{B}^{2}}{{\left|1+\sum_{k=1}^{N}a_{k}e^{-i2\pi kT_{s}}\right|}^{2}}$$

where *f* ∈ [0~*f_s_*/2] (generally *f* ∈ [0~*f_s_*/2.56]) and *T_s_* = 1/*f_s_*.

## 3. Experiment

### 3.1. Experimental Bench

**Figure 6:**
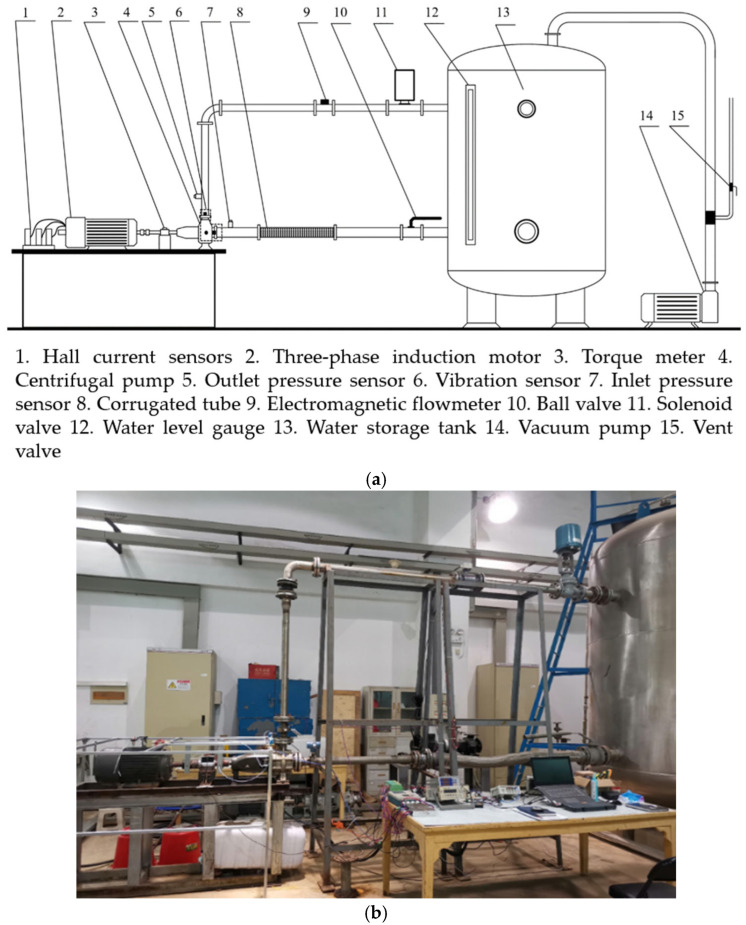
Experimental bench: (**a**) schematic diagram and (**b**) photograph.

A pump performance testing platform is established utilizing a LabVIEW-based signal acquisition system to collect vibration, flowrate, inlet/outlet pressure, torque, and rotational speed signals under various operating conditions. Figure 6a presents the schematic diagram, and Figure 6b shows the physical setup of the experimental bench. The water circulation system consists of centrifugal pumps, stainless steel pipelines, bellows, solenoid valves, a water storage tank, and other auxiliary components. This study uses an IS-65-50-160 single-stage single-suction bedroom centrifugal pump, and its design parameters are shown in Table 1.

**Figure 7:**
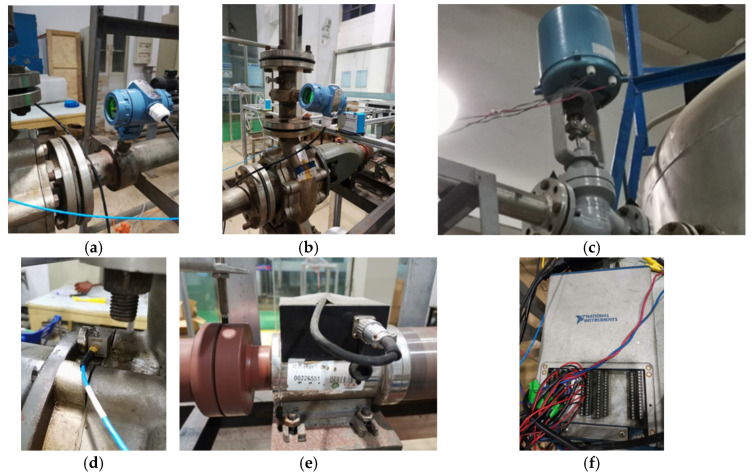
Sensors and the acquisition card used in the experiment: (**a**) inlet pressure sensor (**b**) outlet pressure sensor (**c**) solenoid valve; (**d**) vibration sensor; (**e**) torque mater; and (**f**) NI USB-6343 acquisition card.

Flowrate, inlet/outlet pressure, torque, and vibration signals of the centrifugal pump are collected using different sensors. The YJ-208 static pressure sensor measures water pressure, while the LDG-SIN-CN65-Z2 electromagnetic flowmeter monitors flowrate. The SGDN-50 dynamic torque sensor records both torque and rotational speed. Vibration signals are collected using CT1020LC IEPE acceleration sensor. All signals are transmitted to a NI USB-6343 data acquisition card. Figure 7 shows the sensors and acquisition card used in the experiment, and Table 2 summarizes their key parameters.

### 3.2. Design of the Experiment

**Figure 8:**
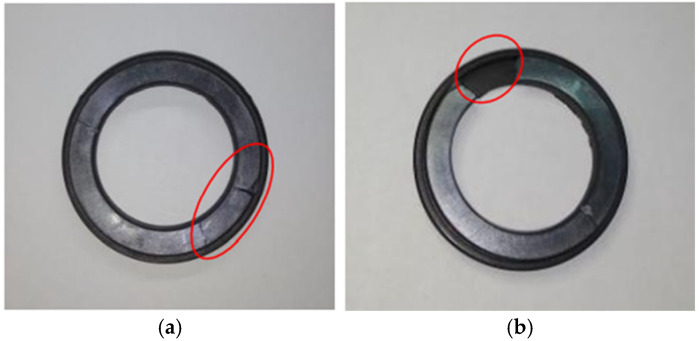
Damaged static ring and face. (**a**) Static ring end face under slight extrusion failure. (**b**) Static ring end face under severe extrusion failure.

In this study, experiments are conducted to measure pump parameters under various rotary-shaft-seal conditions and to assess how these conditions influence pump performance. The experimental procedure for evaluating the external performance characteristics of the pump under normal rotary-shaft-seal conditions is as follows:

1. Fully open the ball valve in the inlet pipeline and ensure that the liquid level pressure in the water storage tank is approximately equal to atmospheric pressure;
2. Turn on the centrifugal pump unit, inspect the hydraulic circulation system for any leaks, verify the stability of the data acquisition card during data collection, and calibrate each sensor;
3. The flow rate of the centrifugal pump is treated as an independent variable, with the rated flow rate *Q_d_* serving as the reference. The full flow range along the horizontal axis is divided into 14 operating points: 0, 0.1*Q_d_*, 0.2*Q_d_*, 0.3*Q_d_*, 0.4*Q_d_*, 0.5*Q_d_*, 0.6*Q_d_*, 0.7*Q_d_*, 0.8*Q_d_*, 0.9*Q_d_*, 1.0*Q_d_*, 1.1*Q_d_*, 1.2*Q_d_*, and 1.3*Q_d_*;
4. Adjust the analog voltage output from the data acquisition card to control the solenoid valve opening, thereby regulating the centrifugal pump’s flowrate. Starting from working point 0, power on the system and initiate signal acquisition once the operating parameters stabilize;
5. Adjust the voltage signal generator to regulate the valve opening, thereby controlling the flow rate. Open the ball valve briefly to purge trapped air, then close it. Once the signals stabilize, begin data acquisition to simultaneously collect inlet and outlet pressure, flow rate, speed, torque, and other key parameters of the centrifugal pump;
6. Repeat step 5 for each of the 14 operating points until data collection is completed for all operation conditions.
7. Using the collected inlet and outlet pressure, flowrate, speed, and torque data under different working conditions, apply Formulas (9)–(14) to compute the corresponding performance parameters. To measure pump performance under mild rotary-shaft-seal failure, first power off the system, remove the standard rotary shaft seal, and replace it with a slightly damaged one (as shown in Figure 8). Then, power on the system and repeat steps 3 to 7.

The head of the centrifugal pump is given as follows:

(9) $$H=Z_{o}-Z_{i}+\frac{P_{o}-P_{i}}{\rho g}+\frac{v_{o}^{2}-v_{i}^{2}}{2g}$$

where *P_i_* and *P_o_* are the static pressure of liquid at the inlet and outlet of the pump. *Z_i_* and *Z_o_* are the distances from the inlet and outlet of the pump to the measuring reference plane. *v_i_* and *v_o_* are the flow velocities at the inlet and outlet of the pump, given as follows:

(10) $$v_{i}=\frac{\pi d_{i}^{2}}{4Q}$$

(11) $$v_{o}=\frac{\pi d_{o}^{2}}{4Q}$$

The pump’s power is given as follows:

(12) $$P=\frac{Tn}{9550}$$

where *T* is torque and *n* is rotational speed.

The output power of a centrifugal pump is given as follows:

(13) $$P_{e}=\frac{\rho gQH}{1000}$$

The efficiency of a centrifugal pump is defined as the ratio of its output power to its input power, given as follows:

(14) $$\eta =\frac{P_{e}}{P}\times 100\%$$

Figure 8a shows the rotary shaft seal with a minor fault. Figure 8b shows the damaged rotary shaft seal.

### 3.3. Experiment Results

**Figure 9:**
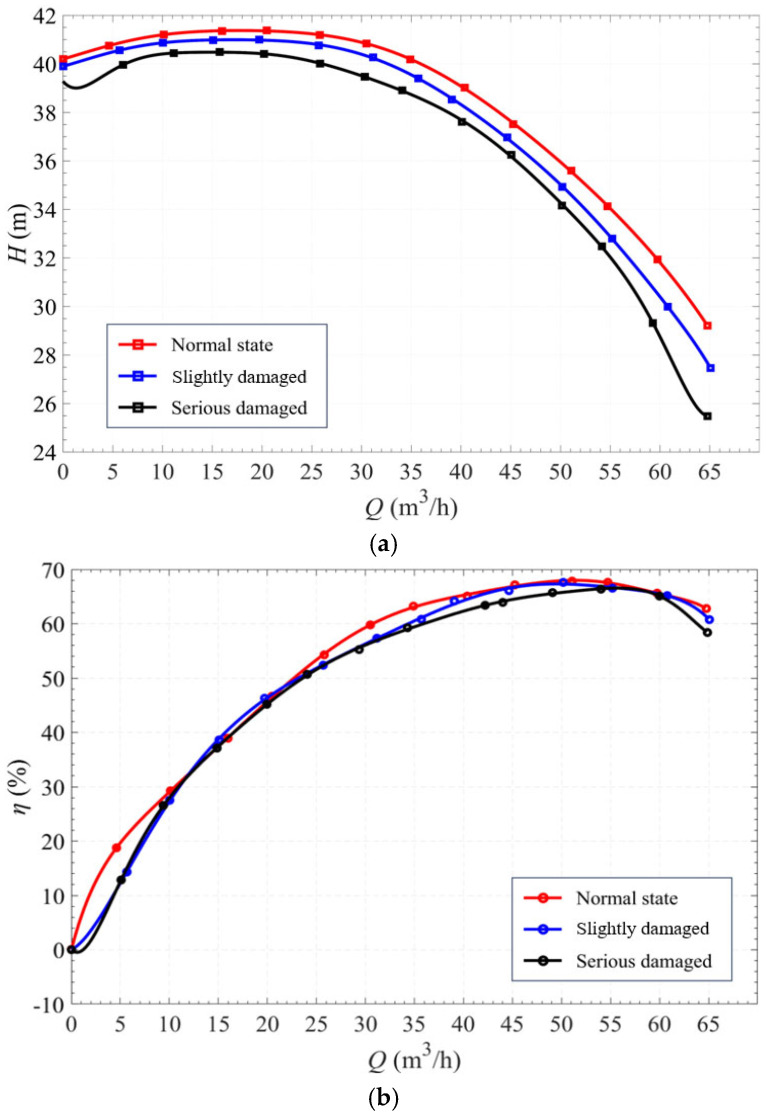

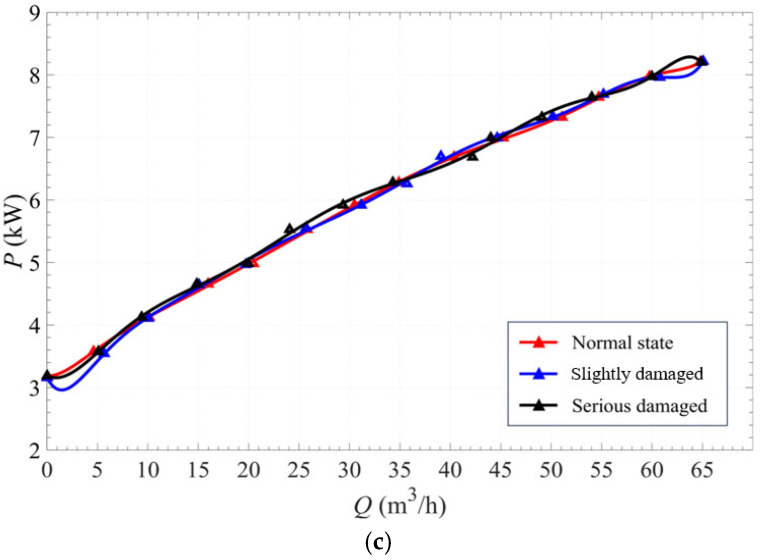
Performance curves: (**a**) *Q*-*H* curves; (**b**) *Q*-*η* curves; and (**c**) *Q*-*P* curves.

The performance curves of the centrifugal pump under different degrees of rotary-shaft-seal faults are shown in Figure 9.

A failure in the rotary shaft seal can lead to serious consequences for the pump system. Initially, fluid leakage occurs, which induces turbulence within the internal flow field. This turbulence causes significant fluctuations in torque. The performance curves show that the rotary-shaft-seal condition has the greatest influence on the pump head. As the severity of the seal fault increases, the head decreases notably. In contrast, efficiency is less sensitive to seal failure, although it still declines with worsening fault conditions. Interestingly, the shaft power remains largely unaffected by changes in the rotary-shaft-seal state.

## 4. Vibration Signals Analysis

Typically, a vibration sensor is mounted on the pump casing near the bearing to capture radial vibration signals [36]. In this study, vibration acceleration signals were collected in three directions: radial, longitudinal, and axial. Among them, the radial signal was selected for further analysis. The experiment collects vibration signals under different rotary-shaft-seal conditions and flowrates. This study conducts a comprehensive analysis from two perspectives:

1. Consistent rotary-shaft-seal conditions with different flow rates;
2. Consistent flowrate with different rotary-shaft-seal states. By processing and analyzing the vibration signals, distinctive and representative indicators can be identified to accurately assess the system’s operational state. The following section presents the signal processing procedure under normal rotary-shaft-seal conditions and standard operating parameters.

### 4.1. Preprocessing

**Figure 10:**
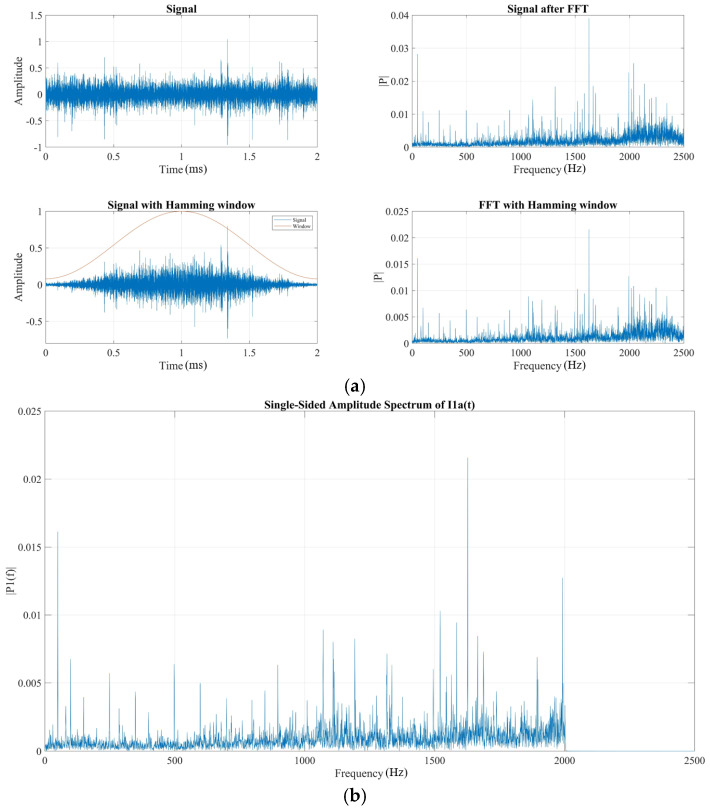
Preprocessing: (**a**) windowed signal and (**b**) anti-aliasing filtered signal.

The sampling frequency of vibration is 5000 Hz. First, the data is normalized so that its amplitude ranges between -1 and +1. A Hamming window is then applied to suppress spectral sidelobes. The sidelobe elevation and decay rate significantly influence spectral energy leakage—higher sidelobes indicate greater leakage, while slower decay results in more pronounced spectral tailing. Conversely, minimal sidelobe energy, approaching zero, yields a more accurate spectral representation. The comparison of signals before and after adding windows is shown in Figure 10a. Then, anti-aliasing filtering is performed on the windowed signal. A low-pass filter is used, with a passband cut-off frequency of 2000 Hz and a stopband attenuation of 74 dB. This filtering removes frequency components above 2000 Hz and enhances the quality of the digital signal. The results after anti-aliasing filtering are shown in Figure 10b.

**Figure 11:**
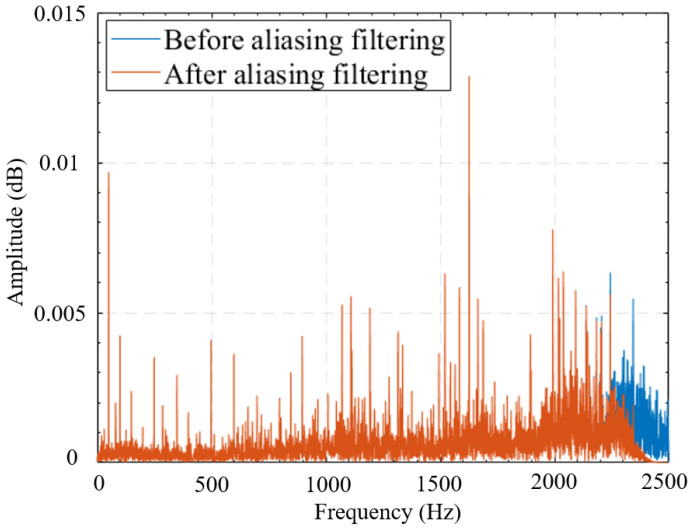
Spectrum comparison between the signals before and after aliasing filtering.

A spectrum comparison between the original and processed signals has been added in the revised manuscript, as shown in Figure 11.

In Figure 11, it can be seen that after anti-aliasing processing, the high-frequency noise interference in the frequency band around 2500 Hz has been partly reduced.

### 4.2. Time–Frequency Joint Analysis

**Figure 12:**
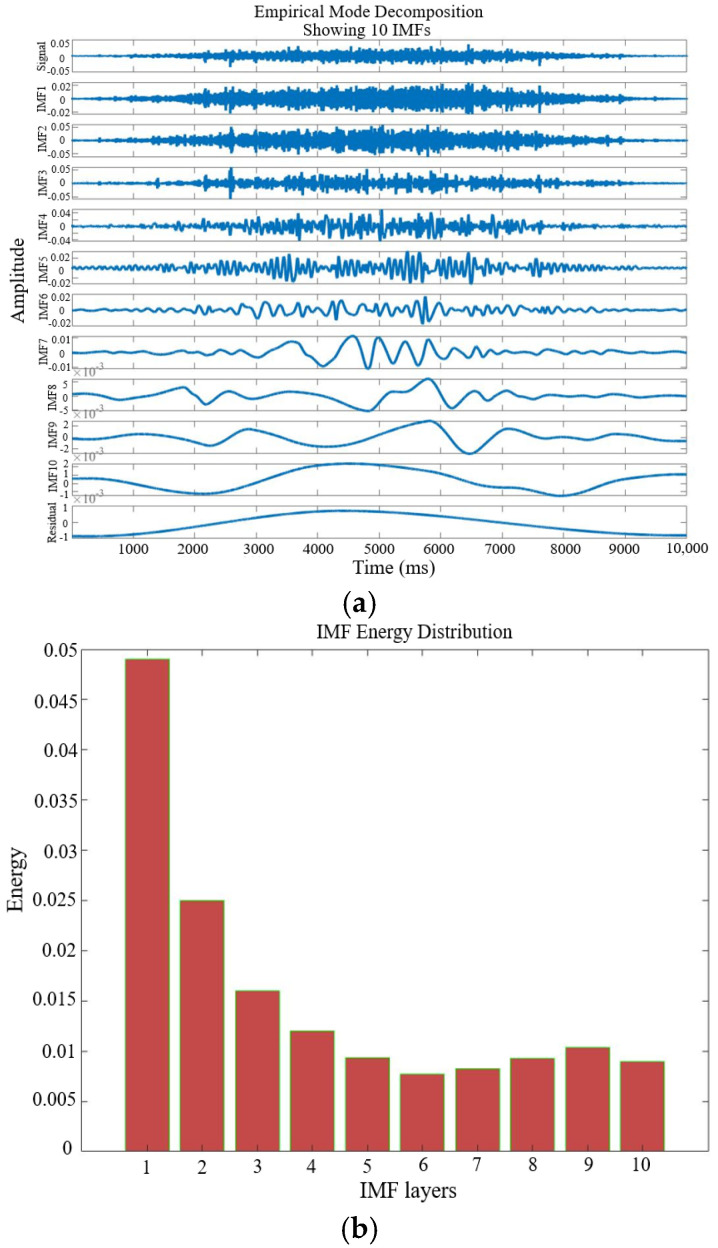

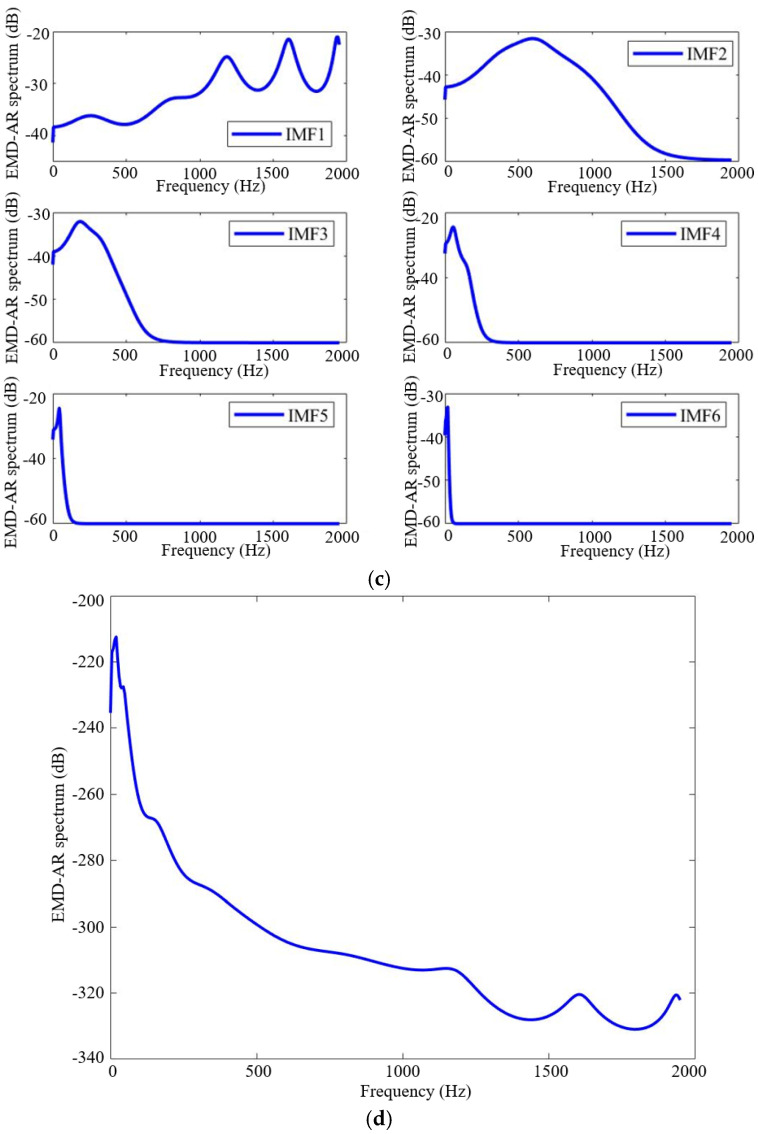
Time–frequency analysis: (**a**) EMD of unsteady vibration signals; (**b**) IMF energy distribution diagram of unsteady vibration signal; (**c**) EMD-AR spectrum of the IMFs; and (**d**) accumulated energy of the EMD-AR spectrum of the first six IMF components.

After signal preprocessing, time–frequency analysis is conducted. This study employs the EMD-AR spectrum method to monitor the condition of the rotary shaft seal. Following windowing and anti-aliasing filtering, the vibration signal is decomposed using Empirical Mode Decomposition (EMD), resulting in several Intrinsic Mode Functions (IMFs), as illustrated in Figure 12a. Based on the energy distribution shown in Figure 12b, the first six IMF components are selected for feature extraction, as they contain the majority of the signal’s energy. Figure 12c presents the EMD-AR spectra of these six IMFs individually, while Figure 12d displays the EMD-AR spectrum of their combined contribution.

**Figure 13:**
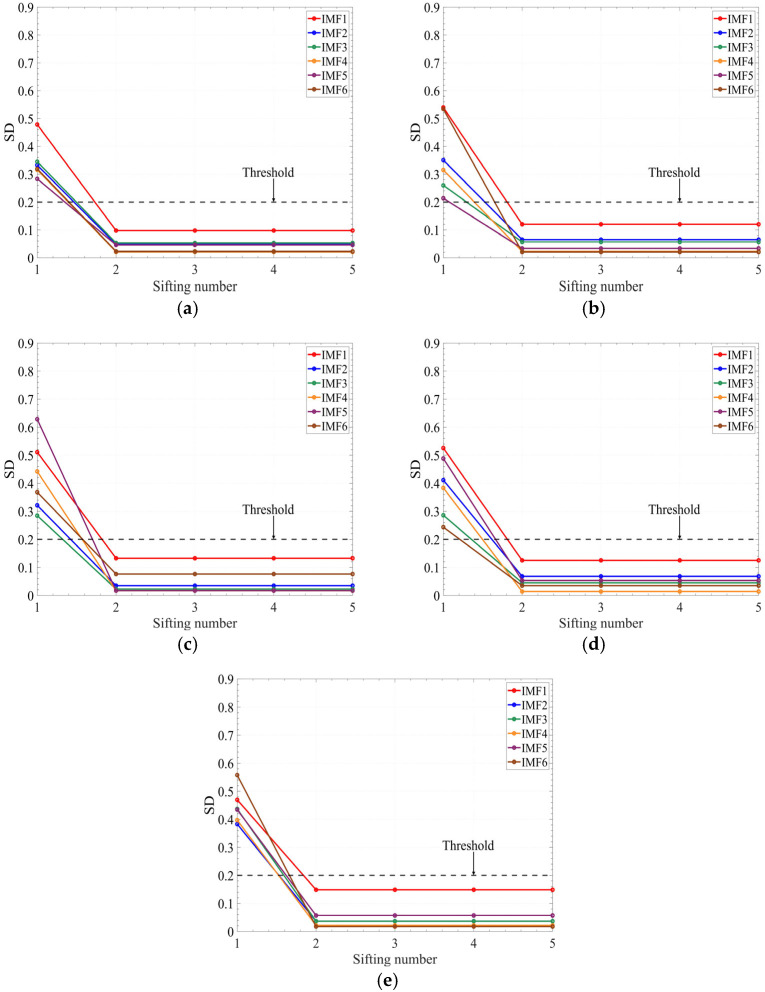
The curves between the sifting number and SD under different flowrates: (**a**) 30 m^3^/h; (**b**) 40 m^3^/h; (**c**) 50 m^3^/h; (**d**) 60 m^3^/h; and (**e**) 70 m^3^/h.

**Figure 14:**
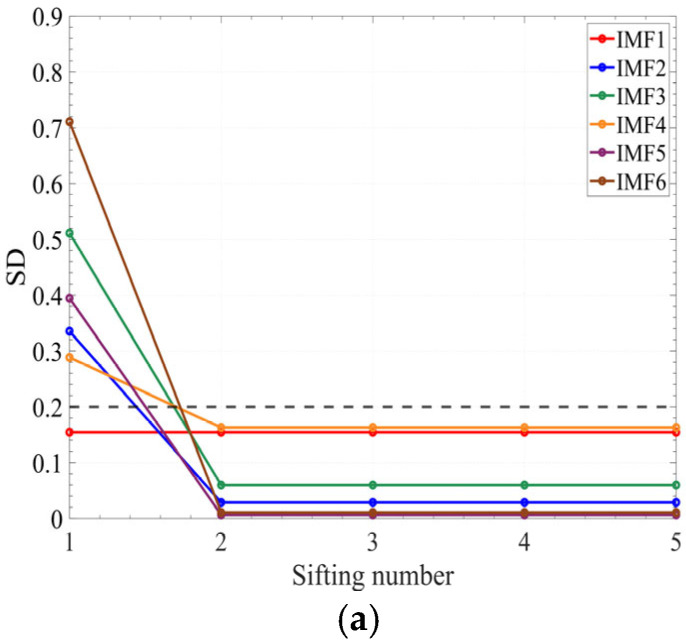

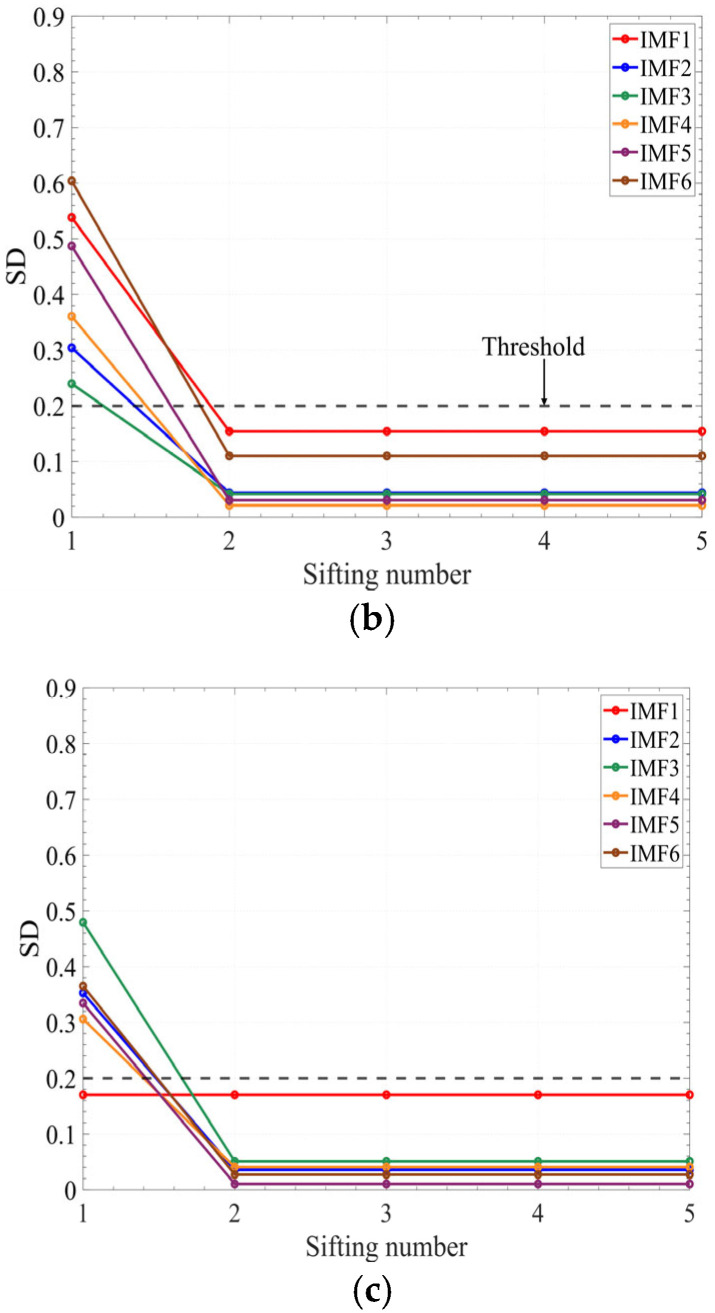
The curves between the sifting number and SD under different fault states: (**a**) small end-face clearance; (**b**) Normal state; (**c**) large end-face clearance.

In this research, the sifting iteration stopping criterion is applicable across all operation conditions. To verify this, the stability analysis of IMF components under different flowrates and different fault states was added in the revised manuscript. Figure 13 shows the curves between the SD and sifting number under different flowrates, Figure 14 shows the curves between the SD and sifting number under different fault states.

In Figure 13, it can be seen that the SD of the first six IMF components under different flowrates decreased below the threshold (0.2) after two iterations. In Figure 14, it can be seen that the SD of the first six IMF components under different fault states at 1.0*Q_d_* also decreased below the threshold after two iterations. Therefore, it can be concluded that the sifting iteration stopping criterion is applicable across all operating conditions.

### 4.3. Analysis Results at Different Flowrates

**Figure 15:**
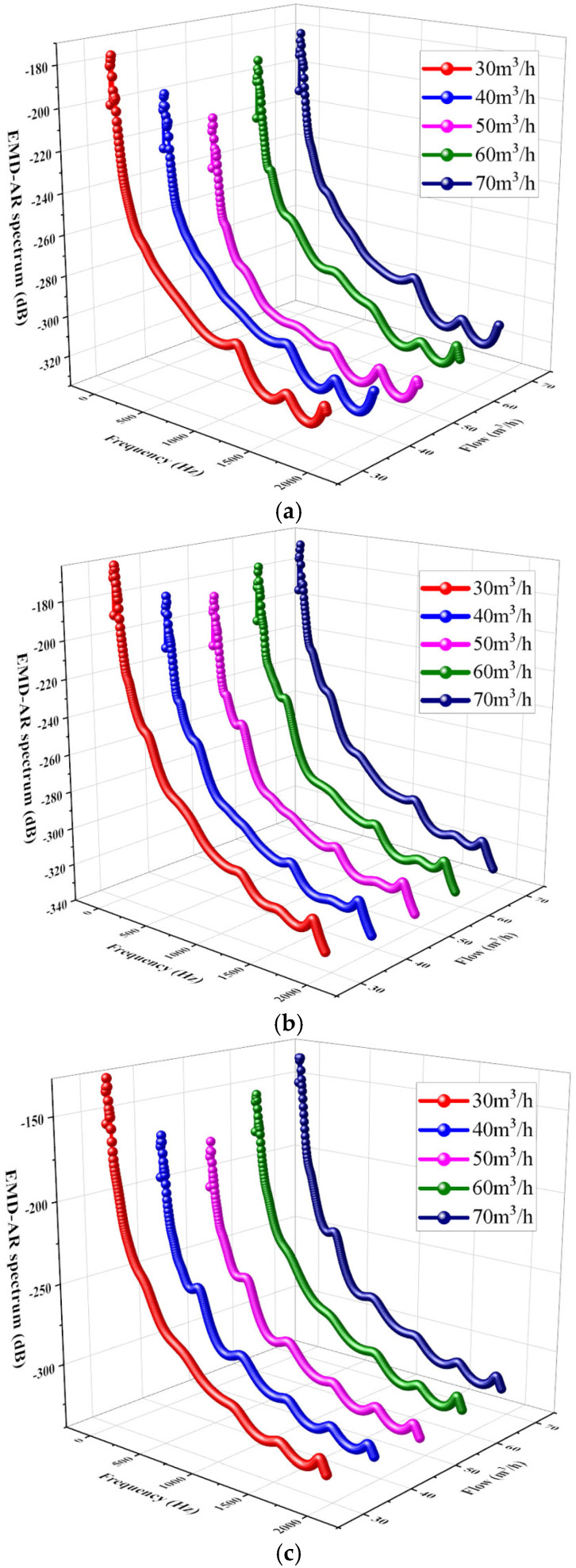
Spectra of EMD-AR under different operational conditions: (**a**) with a standard rotary-shaft-seal state; (**b**) under slight rotary-shaft-seal failure; and (**c**) under serious rotary-shaft-seal failure.

Vibration signals recorded at different flow rates (0.6*Q_d_*, 0.8*Q_d_*, 1.0*Q_d_*, 1.2*Q_d_*, 1.4*Q_d_*) under normal rotary-shaft-seal conditions were analyzed using the EMD-AR spectrum method, as shown in Figure 15a. For comparison, the EMD-AR spectra under mild and severe rotary-shaft-seal fault conditions are presented in Figure 15b,c.

Figure 15 shows that, at 2900 r/min, the dominant concentration of power in the EMD-AR spectrum is located below 500 Hz. The EMD-AR spectral amplitude reaches the lowest at 50 m^3^/h, which means the EMD-AR spectral energy is lowest, corresponding to minimal vibration and enhanced system stability at *Q_d_*. At 40 m^3^/h and 60 m^3^/h, the spectral energy increases slightly but remains relatively low. At 30 m^3^/h and 70 m^3^/h, the signal energy increases significantly. The EMD-AR spectrum offers a more precise representation of pump stability based on vibration characteristics. At a constant flow rate, the EMD-AR spectral amplitude increases with the severity of rotary-shaft-seal degradation: from standard, to minor failure, to serious failure. This observation indicates that vibration amplitude increases proportionally with the severity of shaft-seal failure. Consequently, the amplitude of the EMD-AR spectrum can serve as a diagnostic indicator for assessing the condition of the pump’s shaft seal.

### 4.4. Analysis Results at Different Rotary-Shaft-Seal States

**Figure 16:**
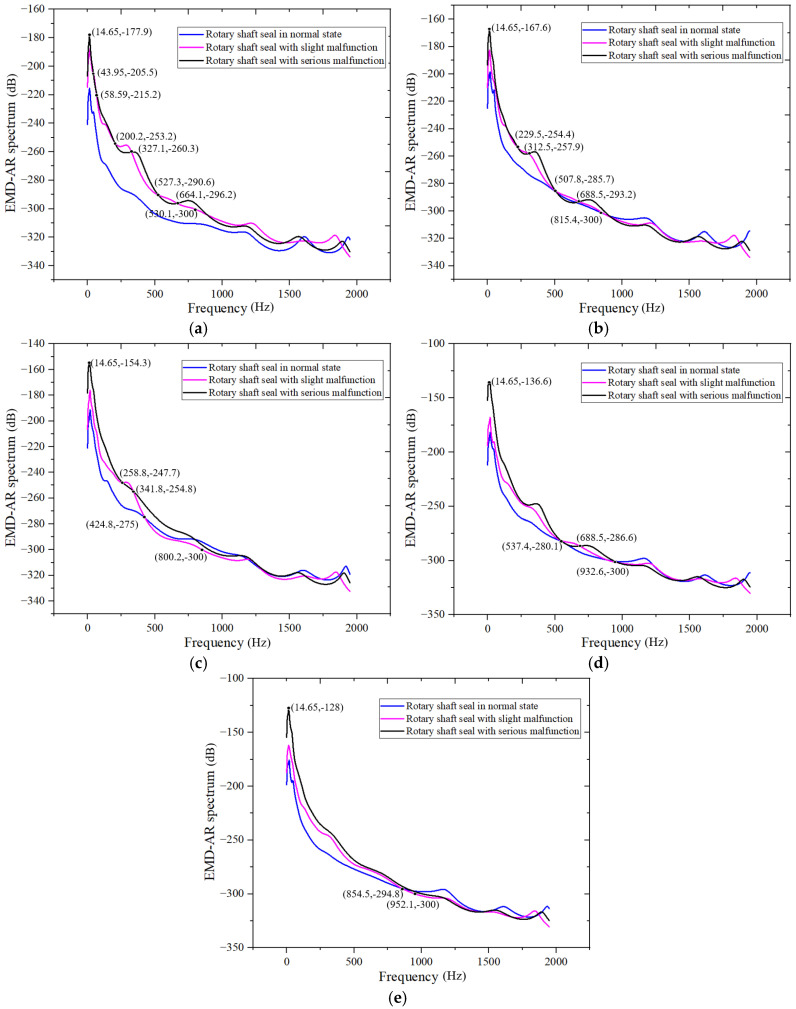
Comparison of the accumulated energy of the EMD-AR spectra of the initial six IMF components under three different rotary-shaft-seal states: (**a**) under *Q* = 50 m^3^/h; (**b**) under *Q* = 40 m^3^/h; (**c**) under *Q* = 60 m^3^/h; (**d**) under *Q* = 70 m^3^/h; and (**e**) under *Q* = 30 m^3^/h.

Figure 16 shows a comparison of EMD-AR spectra under three rotary-shaft-seal states at *Q* = 50, *Q* = 40, *Q* = 60, *Q* = 70, and *Q* = 30 m^3^/h operating conditions.

As shown in Figure 16a, the dominant power amplitude energy in the EMD-AR spectrum is primarily concentrated in the low-frequency range (−300 dB is the critical point), specifically below 530 Hz. As the severity of the rotary-shaft-seal failure increases, the amplitude near the spectral peak at approximately 15 Hz rises accordingly. This trend indicates that this frequency band is highly sensitive to shaft seal degradation. The characteristic frequency bands where EMD-AR energy increases from normal to minor and severe shaft seal failures are approximately 0–45 Hz, 60–200 Hz, and 328–528 Hz. These bands serve as potential diagnostic indicators for identifying rotary-shaft-seal faults.

The analysis approach for Figure 16b–e is consistent with that used in Figure 16a. Table 3 summarizes the dominant frequency bands of energy concentrations in the EMD-AR spectra and the characteristic frequency bands where amplitude increases with the worsening of rotary-shaft-seal failure under each operating condition. Based on the intersection of the frequency bands across different conditions, the final characteristic frequency ranges that can effectively assess the severity of rotary-shaft-seal failure are identified as 0–45 Hz, 60–200 Hz, and 342–425 Hz. In general, as the degree of rotary-shaft-seal failure increases, the EMD-AR spectral amplitude also rises, indicating intensified vibration. This phenomenon is attributed to leakage caused by seal failure, which alters the internal flow within the pump and leads to fluctuations in hydraulic load torque. These fluctuations disturb the force balance of the rotor shaft system, increasing the amplitude of torque oscillations and, consequently, the overall vibration of the centrifugal pump system. The EMD-AR spectrum provides smooth and high-resolution results across a wide frequency range, enabling the reliable identification of shaft-seal faults. It can therefore serve as a useful diagnostic tool for assessing the severity of rotary-shaft-seal damage.

**Figure 17:**
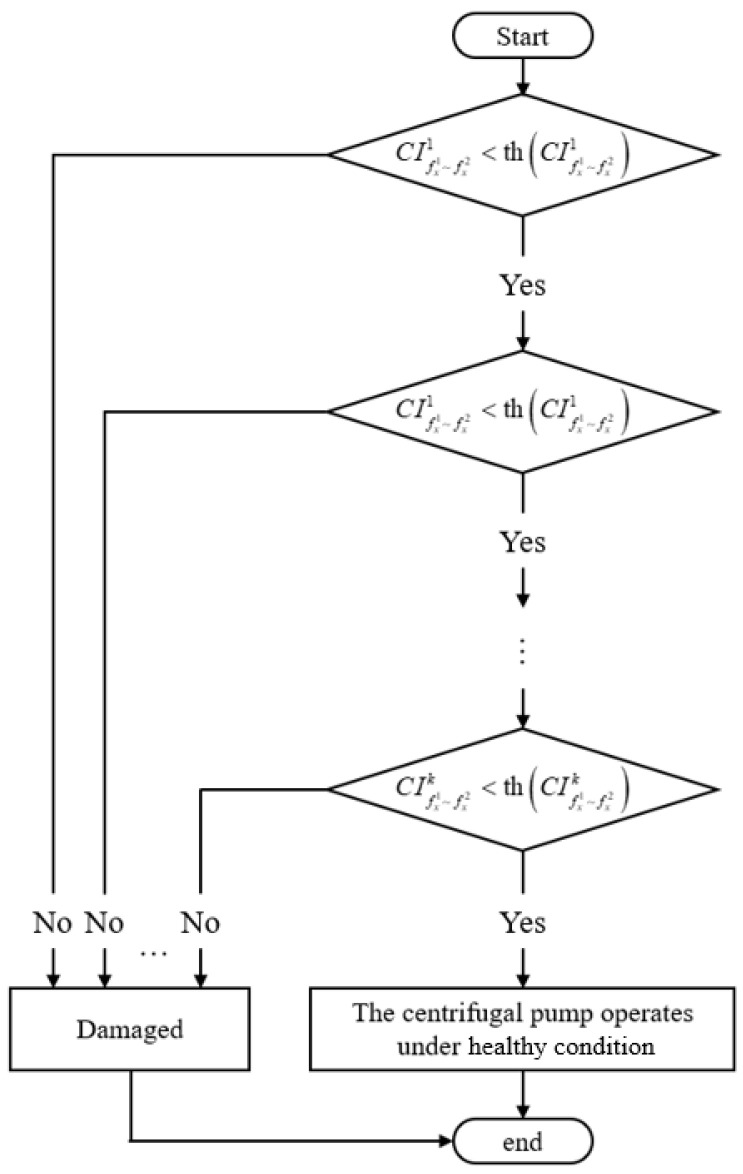
The diagnosis process based on characteristic frequency bands.

In this research, the characteristic frequency bands are selected to evaluate the fault degree of the centrifugal pump under corresponding flowrates. This is because the amplitude of EMD-AR spectra within these bands would monotonically increase as the damage of rotary shaft seal gets worse, whereas in other frequency ranges, the amplitude variations of the EMD-AR do not exhibit such a regular pattern. In the revised manuscript, condition indicators (*CI*) are built for determining whether a fault has occurred, given by the following:

(15) $$CI_{f_{x}^{1}∼f_{x}^{2}}^{k}=\frac{\Delta f\cdot \sum_{f=f_{x}^{1}}^{f_{x}^{2}}{(\mathrm{EMD-AR}}_{f})}{f_{x}^{2}-f_{x}^{1}}$$

where $f_{x}^{1}$ is the lower limit of the *k*th characteristic frequency band, $f_{x}^{2}$ is the upper limit of the *k*th characteristic frequency band, and Δ*f* is the frequency resolution. In this research, we averaged 441 sets of samples of *CI* calculated under normal states as threshold, given by the following:

(16) $$\mathrm{th}\left(CI_{f_{x}^{1}∼f_{x}^{2}}^{k}\right)=\sum_{i=1}^{n}\frac{\Delta f\cdot \sum_{f=f_{x}^{1}}^{f_{x}^{2}}{(\mathrm{EMD-AR}}_{f})}{f_{x}^{2}-f_{x}^{1}}$$

where th(∙) represents the threshold calculation formula and *n* is the number of samples under normal states; in this research n is 441. The diagnosis process based on characteristic frequency bands is shown in Figure 17.

In this research, statistical analyses of sensitivity and specificity have been conducted. The calculation methods of sensitivity (TPR) and specificity (SPC) are given by the following:

(17) $$\mathrm{TPR}\;=\;\frac{\mathrm{TP}}{\mathrm{TP}\;+\;\mathrm{FN}}$$

(18) $$\mathrm{SPC}=\frac{\mathrm{TN}}{\mathrm{TN}+\mathrm{FP}}$$

where TP is true positive, FN is false negative, TN is true negative, and FP is false positive. A total of 230 condition indicators under damaged conditions and 230 condition indicators under healthy conditions were calculated under each flowrate. The statistical measure results of sensitivity and specificity are shown in Table 4.

In Table 4, it can be seen that the sensitivity and specificity under different flowrates are all above 90%. This indicates that the proposed method based on characteristic frequency bands of EMD-AR spectrum basically meets the fundamental requirements for fault diagnosis of centrifugal pumps.

## 5. Conclusions

This study investigates the vibration characteristics of a system under various rotary-shaft-seal conditions and operating states through controlled experiments. Vibration signals corresponding to different rotary-shaft-seal conditions were collected and analyzed using normalization, windowing, anti-aliasing filtering, and EMD-AR spectrum analysis. The following conclusions are drawn:

1. The impact of rotary-shaft-seal failure on key performance indicators follows a descending order of sensitivity: head, efficiency, and shaft power. With increasing severity of rotary-shaft-seal failure, both the head and efficiency exhibit a declining trend, whereas the shaft power remains relatively unchanged. These findings suggest that shaft seal failures result in internal fluid leakage, leading to unsteady flow patterns and non-uniform loading on the impeller and blades. As a result, the overall hydraulic and mechanical performance of the pump is adversely affected.
2. The vibration signals are preprocessed as follows: First, the original vibration signal is normalized to a range between −1 and +1, which eliminates scale differences and enhances both comparability and interpretability of the data, thereby facilitating more effective analysis and model development. Next, a window function is applied to concentrate the signal’s energy in the main lobe, thereby reducing spectral leakage. Finally, an anti-aliasing filter is applied to suppress noise and interference outside the useful signal bandwidth.
3. After preprocessing, time–frequency analysis is conducted on the vibration signal. First, EMD is applied to the signal to extract IMF components at each level. The first six IMF components, representing the primary oscillatory modes of the non-stationary vibration signal, are selected to construct their corresponding EMD–autoregressive (EMD-AR) spectra.
4. Under identical rotary-shaft-seal conditions, when the centrifugal pump operates within its specified design parameters, the amplitude of the EMD-AR spectrum is at its lowest. This indicates minimal vibration and suggests that the system is relatively stable. When operating conditions deviate slightly from the design point, the spectral energy increases slightly compared to the design condition, but the rise is not substantial. In contrast, a significant deviation from the design condition leads to a marked increase in vibration energy, indicating intensified vibrations and reduced system stability. It is particularly important to avoid operating the pump under low-flow conditions. Instead, operation should be maintained at or slightly above the design flow rate to ensure minimal vibration and improved system stability. The EMD-AR spectrum offers a more accurate representation of the operational stability of centrifugal pumps based on vibration signal analysis.
5. Under identical flow rate conditions, the EMD-AR spectrum demonstrates smooth and well-defined characteristics with high spectral resolution. It effectively identifies rotary-shaft-seal faults across a broad frequency range and serves as a reliable diagnostic indicator for fault detection. Experimental data analysis reveals that the power amplitude energy of the EMD-AR spectrum under rotary-shaft-seal faults is predominantly concentrated within the low-frequency range below 1000 Hz. Specifically, the frequency bands of 0–45 Hz, 60–200 Hz, and 342–425 Hz are identified as characteristic bands that enable effective diagnosis of the severity of rotary-shaft-seal failures.

## Figures and Tables

**Table 1:** Design parameters of the centrifugal pump.

Parameter	Symbol (Unit)	Value
Impeller inlet diameter	*D*1 (mm)	74
Impeller outlet diameter	*D*2 (mm)	174
Blade outlet width	*b*2 (mm)	12
Number of blades	*Z*	6
Rated flow	*Q_d_* (m^3^/h)	50
Rated head	*Hd* (m)	34
Rated speed	*N* (r/min)	2980
Rated efficiency	η (%)	72.8

**Table 2:** Parameters of the sensors used in the experiment.

Sensor	Parameter	Value
YJ-208 static pressure sensor	Range	0–1.0 MPa
	Output signal	4–20 mA
	Measurement accuracy	0.5%
JDG-SIN-CN65-Z2 electromagnetic flowmeter	Range	0–100 m^3^/h
	Output signal	4–20 mA
	Measurement accuracy	0.5%
	Supply voltage	AC 220 V
CT1020LC acceleration transducer	Sensitivity	200 mV/g
	Range	25 g
	Bandwidth resolution	0.25 mg
SGDN-50 dynamic torque sensor	Output frequency	5–15 kHz
	Measurement accuracy	0.3%
NI USB-6343 multifunctional data acquisition card	Analog output signal	32 channels, 16-bit resolution
	Analog input signal	4 channels, 16-bit resolution
	Input signal voltage range	−10–10 V
	Digital I/O interface	48 channels

**Table 3:** EMD-AR spectra energy concentration frequency band and mechanical seal fault characteristic frequency band under various operating conditions.

*Q*	Frequency Band of Energy Concentration	Characteristic Frequency Band
50 m^3^/h	0–530 Hz	0–45 Hz, 60–200 Hz, 328–528 Hz
40 m^3^/h	0–816 Hz	0–230 Hz, 313–508 Hz, 689–816 Hz
60 m^3^/h	0–800 Hz	0–259 Hz, 342–425 Hz
70 m^3^/h	0–933 Hz	0–538 Hz, 689–933 Hz
30 m^3^/h	0–952 Hz	0–855 Hz

**Table 4:** Sensitivity and specificity under different flowrates based on condition indictors.

*Q*	TPR	SPC
50 m^3^/h	94.3%	97.8%
40 m^3^/h	95.0%	98.3%
60 m^3^/h	93.5%	96.2%
70 m^3^/h	93.8%	97.0%
30 m^3^/h	95.4%	97.4%

## Data Availability

Data is unavailable due to privacy.
